# Design and Experimental Research of Robot Finger Sliding Tactile Sensor Based on FBG

**DOI:** 10.3390/s22218390

**Published:** 2022-11-01

**Authors:** Guan Lu, Shiwen Fu, Yiming Xu

**Affiliations:** 1 School of Mechanical Engineering, Nantong University, Nantong 226019, China; 2 School of Electrical Engineering, Nantong University, Nantong 226019, China

**Keywords:** fiber Bragg grating, sliding tactile sensing, robot finger

## Abstract

Aiming at the problem of flexible sliding tactile sensing for the actual grasp of intelligent robot fingers, a double-layer sliding tactile sensor based on fiber Bragg grating (FBG) for robot fingers is proposed in this paper. Firstly, the optimal embedding depth range of FBG in the elastic matrix of polydimethylsiloxane (PDMS) was determined through finite element analysis and static detection experiments of finger tactile sensing. Secondly, the sensor structure is optimized and designed through the simulation and dynamic experiments of sliding sensing to determine the final array structure. Thirdly, the sensing array is actually pasted on the surface of the robot finger and the sensing characteristics testing platform is built to test and analyze the basic performance of the sliding tactile sensor. Then, the sensor array is actually attached to the finger surface of the robot and the sensing characteristics testing platform is built to experiment and analyze the basic performance of the sliding tactile sensor. Finally, a sliding tactile sensing experiment of robot finger grasping is conducted. The experimental results show that the sliding tactile sensor designed in this paper has good repeatability and creep resistance, with sensitivities of 12.4 pm/N, 11.6 pm/N, and 14.5 pm/N, respectively, and the overall deviation is controlled within 5 pm. Meanwhile, it can effectively sense the signals of the robot fingers during static contact and sliding. The sensor has a high degree of fit with the robot finger structure, and has certain application value for the perception of sliding tactile signals in the object grasping of intelligent robot objects.

## 1. Introduction

With the continuous progress of robot technology, robot plays an important role in the process of intelligent and integrated industrial production. In practical application, sliding signal detection in robot perception is also important [1,2,3]. When grasping an object, the robot needs to obtain information such as whether the object is sliding and the sliding distance for facilitating real-time adjustment of the grasping posture, in order to avoid the damage or dislodgement of the gripped object due to misjudgment of the gripping force value, so as to achieve high-precision grasping and flexible clamping force control [4]. Therefore, it is necessary to conduct more in-depth research on the sliding tactile sensing technology of intelligent robots.

At present, the sensors selected in the research of sliding tactile sensing can be divided into piezoresistive, piezoelectric, capacitive, and optical types according to principle. Among them, the sliding tactile sensor is mainly composed of electrical components, but its flexibility is insufficient [5,6,7]. At the same time, due to the influence of electromagnetic interference, temperature drift, large volume, and other factors, there is a certain degree of accuracy error in the sensing performance, which is difficult to meet the high-precision detection requirements of sliding tactile sensing information.

With the continuous development of fiber sensing technology, fiber Bragg grating (FBG) is more and more widely used in the field of sensing [8,9,10,11,12,13], which has irreplaceable advantages. First of all, optical fiber is made from silica, which has high sensitivity and accuracy, corrosion, and high temperature resistance. At the same time, its small size, light weight, small space occupation, and easy fabrication make it possible to be embedded without affecting the packaging material structure, which is conducive to the integration and miniaturization of sensing elements [14]. In addition, optical signal transmission can effectively ensure the stability of the transmitted signal [15,16]. Fiber grating has a wide range of measurement fields, and can be absolutely measured after calibration. Wavelength division multiplexing (WDM) technology can be used to form a distributed sensor array with high signal-to-noise ratio (SNR), good repeatability, and wide output linear range. Therefore, it is an important research direction to apply FBG sliding tactile sensing technology to the field of intelligent perception at present. In 2018, Qian M. et al. designed a FBG sliding sensor based for mechanical fingers, which can determine the sliding direction based on the wavelength characteristics of each FBG sensor. It enables the mechanical finger to grasp objects easily [17]. In 2019, Jiang Q. et al. designed a sliding sensor for the first knuckle of robot finger [18]. The overall structure is divided into the contact area and the measurement and perception area, which can effectively detect and recognize the sliding signal. In 2022, Sun S. et al. designed a two-layer “cruciform” two-dimensional distributed sensor array [19], which can effectively analyze and distinguish sliding signals.

To sum up, the research on FBG sliding tactile sensor is mainly focused on the single measurement of tactile signal and sliding signal. Meanwhile, due to the large structure size, there are few sliding tactile sensors that can be practically applied to robot fingers.

Therefore, a FBG sliding tactile sensor for mechanical finger is designed in this paper. Polydimethylsiloxane (PDMS) is used as the elastic matrix, which has good flexibility and fit, and can simultaneously detect static contact and sliding signals of the robot finger in the actual grasping process. The research of this paper can provide certain theoretical and experimental basis for the research of robot sliding tactile sensor, and has important meaning for the research and application of FBG sliding tactile sensor in robot intelligent perception.

## 2. FBG Pressure Tactile Sensing Principle

**Figure 1:**
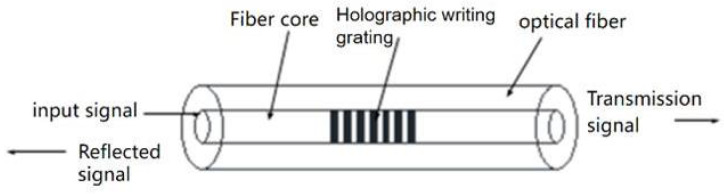
Schematic diagram of FBG.

The FBG sensor is characterized by the periodic refractive index distributed on the fiber core. The principle is to form a phase grating in the fiber core by using the method of interaction between germanium ions in the fiber core and external incident photons. The structure diagram and reflection transmission characteristic diagram of FBG are shown in Figure 1.

According to the analysis of coupled membrane theory, during the transmission of incident light inside the FBG, the FBG will produce coherent reflection of the broadband light that meets the relative frequency and incident conditions in the incident light, forming the central reflection peak [20,21]. The central wavelength of the FBG can be obtained from the equation:

(1) $$\lambda_{B}=2n_{eff}\Lambda$$

In the formula, $\lambda_{B}$ is the reflection wavelength of FBG, $n_{eff}$ is the effective refractive index of optical fiber core, and Λ is the grating spacing. According to Formula (1), the FBG central wavelength $\lambda_{B}$ is highly correlated with grating spacing Λ and core refractive index $n_{eff}$ [22]. Among many physical variables, strain and temperature are the two most important physical variables that affect the FBG wavelength reflection. Therefore, the above equation can also be expressed by strain ε and temperature T:

(2) $$\lambda_{B}=2n_{eff}\Lambda =2n_{eff}\left(\varepsilon ,T\right)\Lambda \left(\varepsilon ,T\right)$$

Strain is the most direct factor affecting the change of FBG center wavelength. Under the condition of constant temperature (△T=0), the FBG shift caused by strain can be expressed by the differential equation:

(3) $$\frac{\Delta \lambda_{B}}{\lambda_{B}}=\left(\frac{1}{n_{eff}}\frac{\partial n_{eff}}{\partial \varepsilon }+\frac{1}{\Lambda }\frac{\partial \Lambda }{\partial \varepsilon }\right)$$

Formula (3) is the strain sensing model of FBG under the condition of constant temperature, indicating that it is theoretically feasible to test sliding tactile sensing signals by using FBG.

## 3. Design of FBG Sliding Tactile Sensing Array for Robot Finger

**Figure 2:**
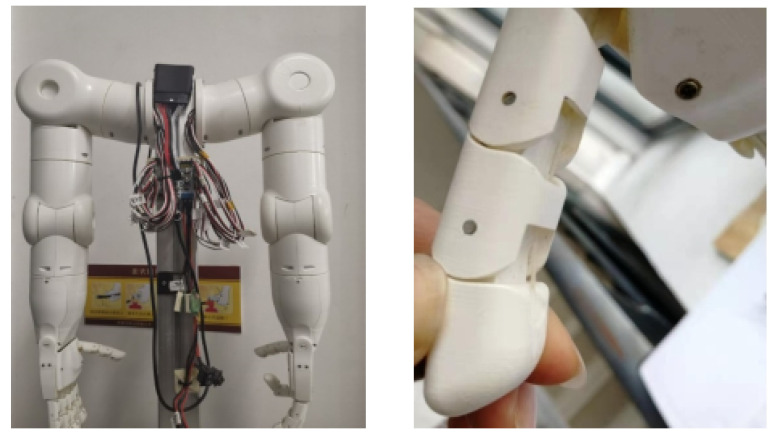
Two-armed robot and fingers.

The robot hand used in this paper is shown in Figure 2. In general, a robot finger contains three knuckles, among which the end knuckle has the closest contact with the outside world [23]. As the main contact surface in the process of object grasping, the inner surface of the robot’s terminal knuckle is a smooth curved surface, which can collect external physical information more comprehensively in the process of contact with objects. Therefore, the inner finger pulp of the end knuckle was selected for the fitting and installation of the sliding tactile sensor, and considering the double-layer structure of the sensor, the size of the sliding tactile sensor can be determined to be about 7 mm × 9 mm × 5 mm.

### 3.1. Simulation and Experimental Analysis of Embedded Depth of Sensing Array Grating Based on Tactile Sensing

**Figure 3:**
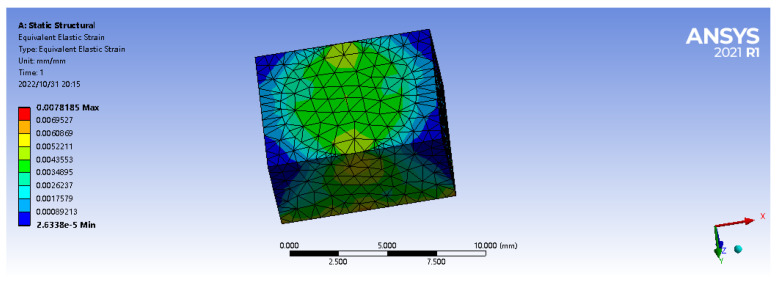
Elastomer simulation model.

In the process of contact between mechanical finger and object, the finger surface will first be subjected to static external load, so it is necessary to improve the sensitivity of FBG inside the elastic body to external load as much as possible, so as to enhance the tactile perception of FBG sensor array. Firstly, Ansys Workbench is used to analyze the optimum embedding depth of elastomer under load. Secondly, an elastic model of 7 mm × 9 mm × 5 mm is established in Geometry. In the material data, the density of the matrix model ρ is set at 1000 Kg/m${}^{3}$, the elastic modulus E is 9.2 MPa, and the Poisson’s ratio is 0.49. The grid division accuracy is set to 0.001 m, as shown in Figure 3.

**Figure 4:**
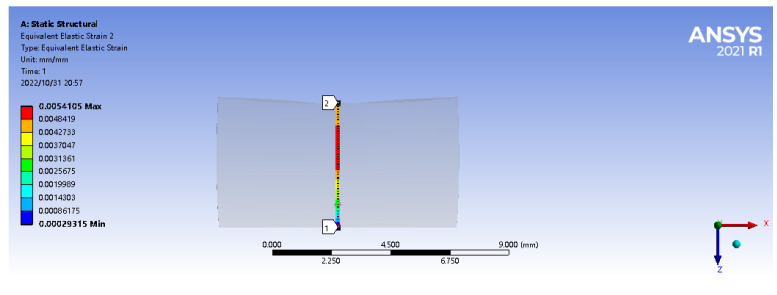
Vertical path diagram.

A vertical path is set at the center point of the matrix model, with the starting point (0,0,0) and the ending point (0,0,0.005). Then a vertical downward 2 N forward concentrated force is applied at the center point (0,0,0.005) of the matrix material’s upper surface. In the practical application process, the matrix material will produce partial displacement in the horizontal direction due to elasticity under the action of load, so a given constraint (displacement) is imposed on the lower surface to the matrix material to force displacement in the horizontal X, Y axis (freedom set to free) and a constraint is applied in the vertical direction Z axis (freedom set to 0), as shown in Figure 4.

**Figure 5:**
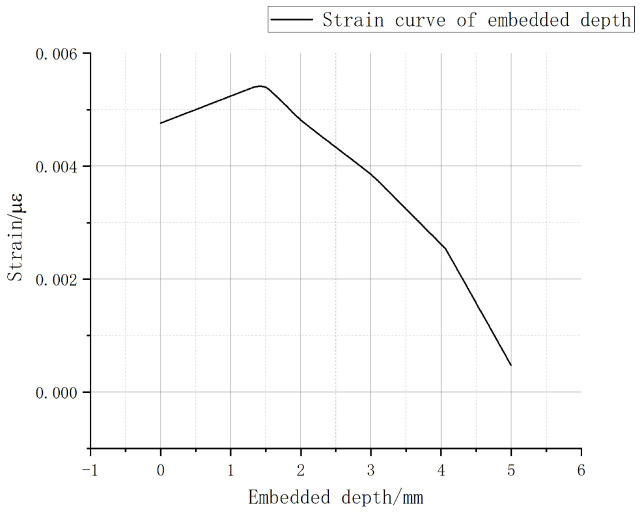
Strain values on the path.

The strain in the path defined above is shown in Figure 5. As can be seen from the figure, under the action of 2 N forward load, when the embedded depth is in the range of 1–5 mm, the strain value is inversely proportional to the embedded depth. The closer to the upper surface, the greater the strain value is. The strain reaches the maximum value of 5411 microstrain at the position 4 mm from the lower surface (the output linear range of fiber grating is wide, and the wavelength shift has a good linear relationship with the strain within the range of 10,000 microstrain). At the same time, within the range of 1 mm to 2 mm from the upper surface, the average strain is the largest, which is the range of the largest strain value.

**Figure 6:**
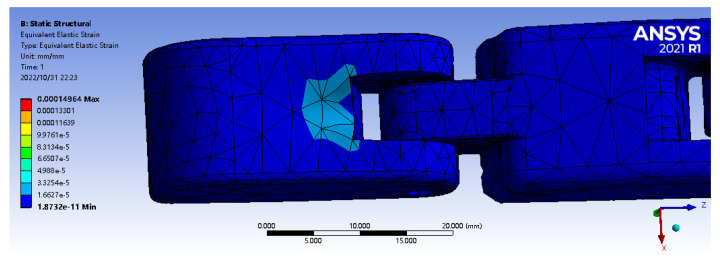
Simulation model of robot finger.

**Figure 7:**
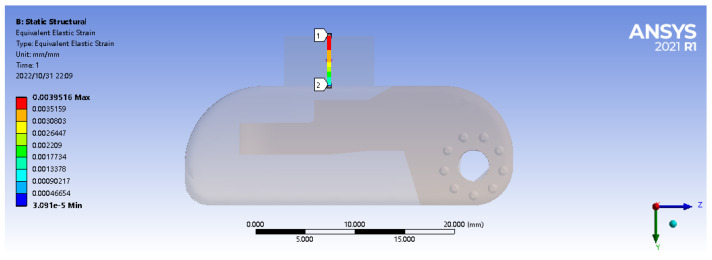
Simulation model of robot finger and elastomer.

Then, in the simulation analysis, the actual contact process before and after the robot finger stick to the matrix was simulated, and the strain simulation analysis was conducted to obtain the optimal embedding depth range of the sensor. The geometry function module is used for 3D modeling, which is imported into the ANSYS Workbench for static simulation analysis of its tactile perception process. The upper surface of finger’s first knuckle is selected as the bonding surface, and the same 7 mm × 9 mm × 5 mm matrix model is established. Set the density of the finger (PVC material) model at 950 Kg/m${}^{3}$, the elastic modulus E at 3.14 GPa, and Poisson’s ratio at 0.42. After 2 N positive load is applied, it can be concluded from the following figure that the median strain of the matrix is 25 times that of the bare finger, and the maximum range of the median strain of the matrix is 0–2 mm from the upper surface (Figure 6 and Figure 7). It shows that the tactile sensor array has played a very good sensing effect, and the optimal embedding depth range of the sensor is consistent with the previous analysis.

In order to verify and obtain the optimal embedding depth of FBG in the process of tactile perception, pressure sensing experiments were carried out for FBG sensors with different embedding depths. Considering the actual embedded level of the encapsulation process (PDMS thickness shall be at least 0.3 mm), the FBG sensors with embedded depths of 0.3 mm, 0.5 mm, 0.8 mm, 1.0 mm, 1.2 mm, and 2.0 mm were fabricated for the pressure sensing experiment. Considering the size of the actual encapsulated elastomer, relevant parameters of FBG selected in this paper are shown in Table 1, and FBG1 and FBG2 are used in the embedding depth experiments.

**Figure 8:**
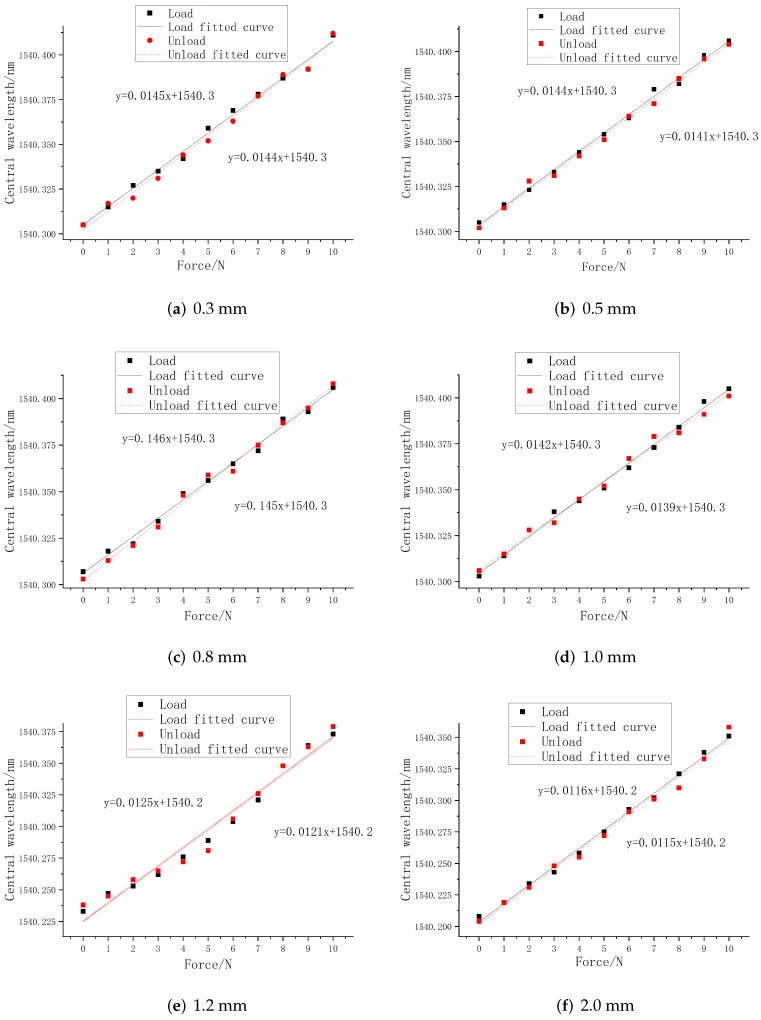
Loading and unloading fitting curve of FBG1.

**Figure 9:**
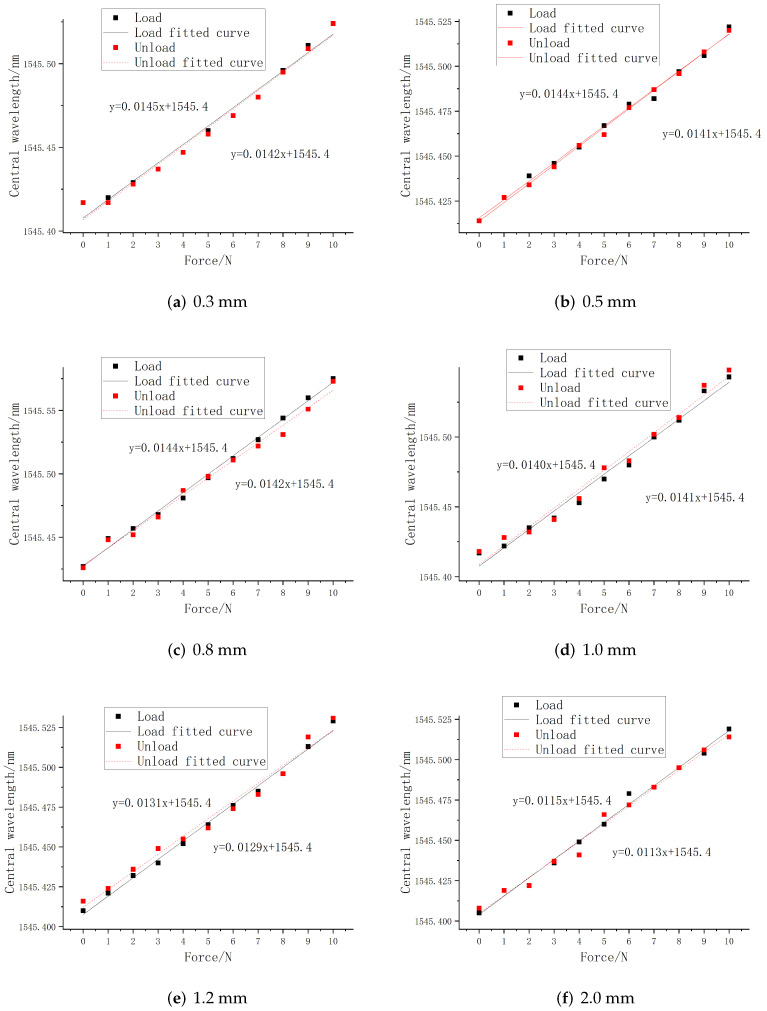
Loading and unloading fitting curve of FBG2.

The external pressure is controlled within the range of 0–10 N, and the step size is set to 1 N. The central wavelength value of each FBG sensor was recorded by the acquisition software of the demodulation equipment, and the curve fitting of central wavelength shift of all sensors with the change of loading force during the loading and unloading process was performed by using Origin software. The linear fitting curves of FBG1 and FBG2 are shown in Figure 8 and Figure 9.

It can be seen from the above figures that under different embedding depths, the central wavelength of FBG sensor has a good linear relationship with the load size, and the overall change is relatively uniform and the central wavelength tends to be linear distribution, indicating that it has a good tactile perception ability. At the same time, according to the linear fitting parameter values in the loading and unloading process obtained in the above figure, Table 2 is drawn below.

The overall variation is relatively uniform and the deviation between the sensitivity of loading and unloading is small. At the same time, the determination coefficient $R^{2}$ of the fit goodness is higher than 98.15%, indicating that PDMS as a packaging material can ensure that the embedded FBG can effectively perceive the external load, and has a linear relationship with the pressure value. The experimental fitting results are consistent with the simulation results, which verifies that it has a good linear sensitivity.

**Figure 10:**
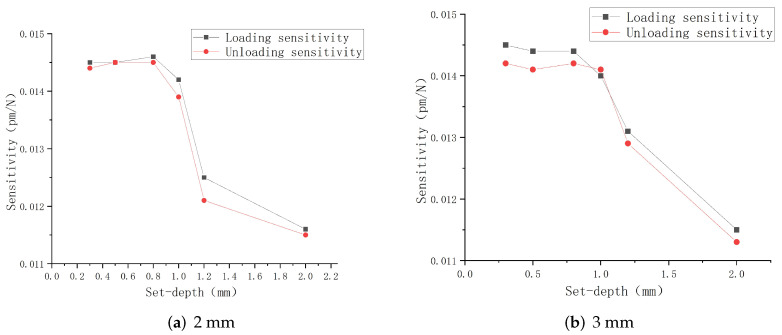
FBG linear sensitivity graph.

According to the fitting parameters in the table, the relationship between the linear sensitivity and the embedded depth is drawn in Figure 10. It can be seen from the figure that the embedded depth will significantly affect the strain sensing degree of the embedded FBG, and determine the central wavelength shift value. Within the range of 1 mm from the upper surface, both FBG1 and FBG2 have the maximum tactile sensitivity, and the mean of the overall linear sensitivity is also the maximum. Comparing the experimental results with the simulation results, it can be seen that in the process of tactile perception, the FBG sensor has the highest tactile perception sensitivity within the range of 1 mm embedded depth, which is basically consistent with the simulation results.

### 3.2. Simulation and Experimental Analysis of Fiber Grating Sensing Array Structure Based on Sliding Perception

When the object slides on the surface of the elastomer, tangential strain will be generated. According to the FBG axial strain sensitivity characteristics, the FBG sliding tactile sensor will identify the sliding state by effectively sensing the tangential strain value in the sliding process. Therefore, the sensing ability of the FBG to the sliding tangential strain value will be crucial. Firstly, in order to obtain the optimal embedded depth of FBG for signal perception during sliding, this section will design and establish a matrix model, and conduct sliding transient simulation analysis on the model.

**Figure 11:**
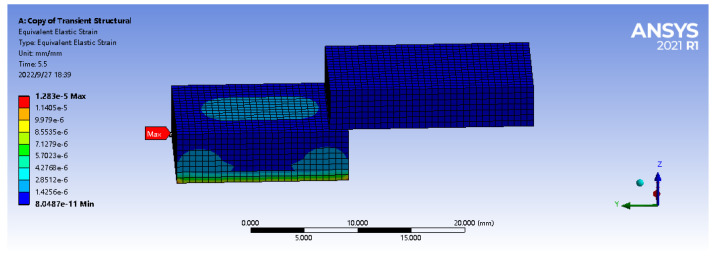
Simulation model of sliding matrix.

As shown in Figure 11, the simulation model of sliding is firstly modeled in Solidworks software. The lower block in the figure is an elastomer matrix model with a size of 7 mm × 9 mm × 5 mm. In the material data, the density of the matrix model ρ is set at 1000 Kg/m${}^{3}$, the elastic modulus E is 9.2 MPa, and the Poisson’s ratio is set to 0.49. The size of the upper block is 6 mm × 11 mm × 4 mm, and the material is set as Aluminium Alloy. The density parameters of the alloy copper block were set to 8300 Kg/m${}^{3}$, the Young’s modulus is 1100 MPa and Poisson’s ratio is 0.34. The model was imported into Ansys Workbench, and the Transient Structural module is selected for the finite element analysis of dynamic contact.

When the multi-part model is imported into the Mechanical module, its contact surface will automatically create bonded contact. Therefore, in connections-contacts, the contact type in Type is changed to frictional contact, and the lower surface of the alloy copper block is set to contact bodies, the upper surface of the elastomer is set to target bodies, and the friction coefficient is set to 0.2. Fixed support is applied to the lower surface of the elastomer, and the standard downward gravity acceleration is applied to the alloy copper block by standard earth gravity.

**Figure 12:**
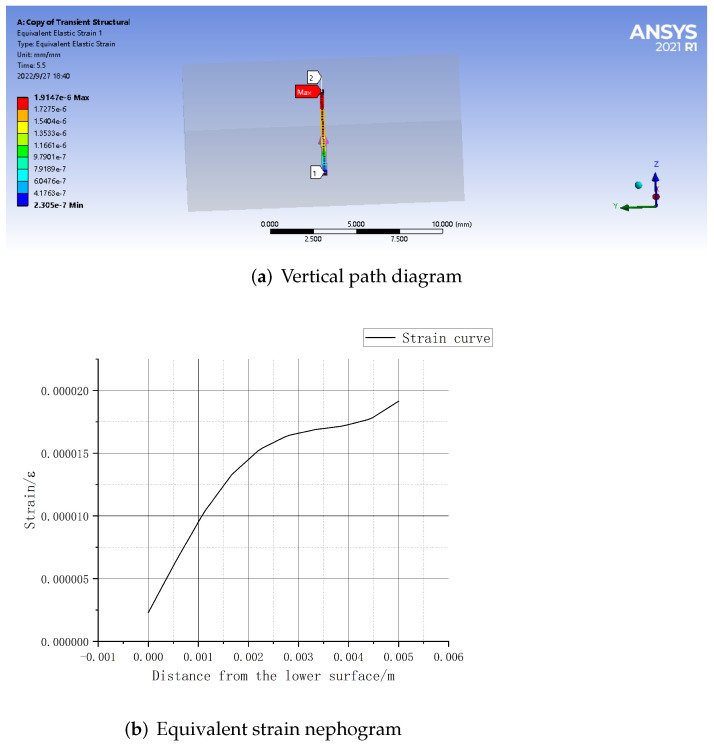
Axial path strain diagram.

As shown in the Figure 12, the elastic body can effectively perceive the tangential strain value generated by the upper surface object in the sliding process. At the same time, in the vertical path along the Z axis, the closer to the upper surface, the greater the sliding equivalent strain value, and there is a proportional relationship between the embedded depth and the strain value. The maximum strain value is $1.9147\times 10^{-6}$, indicating that the FBG sensor based on PDMS can effectively capture the strain signal of the object in the sliding process, and is feasible and reasonable for object sliding detection.

#### 3.2.1. Experimental Analysis of Dynamic Detection of Embedding Depth

**Figure 13:**
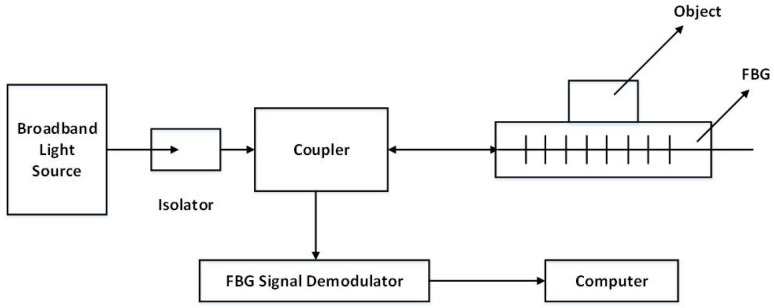
Schematic diagram of the dynamic detection system.

**Figure 14:**
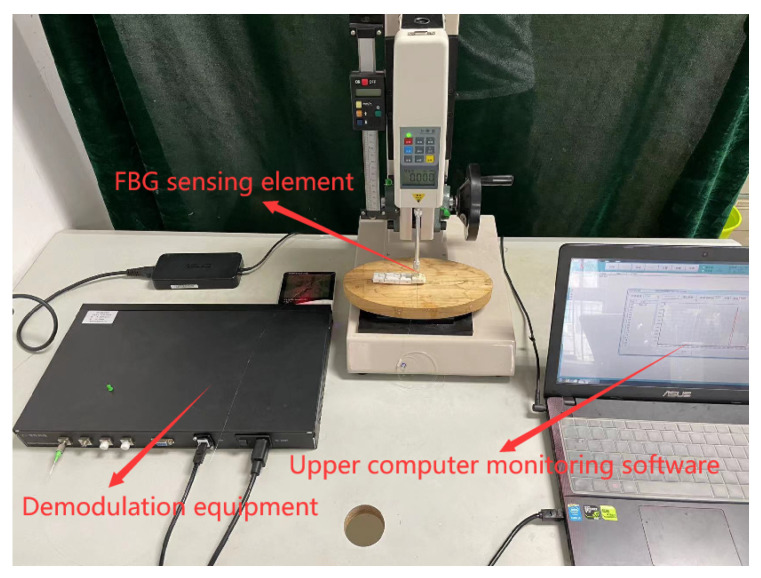
Sliding detection experimental platform.

Sliding sensing experiments were conducted on the three FBG, respectively. After the sensors were encapsulated, paste it on the platform surface to make the sensor fixed. Then, place the copper block on the upper surface of the sensor, paste sandpaper on the surface to increase friction to improve the sensing sensitivity of the FBG to the sliding signal. One end of the sensor was connected to the intelligent drive trolley. Using the intelligent drive trolley to pull the copper block at a constant speed for sliding experiments, until the copper block is completely free from the sensor surface, sliding sense dynamic detection system device schematic diagram is shown in Figure 13. The dynamic detection platform of the sliding sensing experiment consists of demodulation equipment, FBG sensing element, intelligent drive trolley and computer, and the physical diagram of the detection platform is shown in Figure 14.

**Figure 15:**
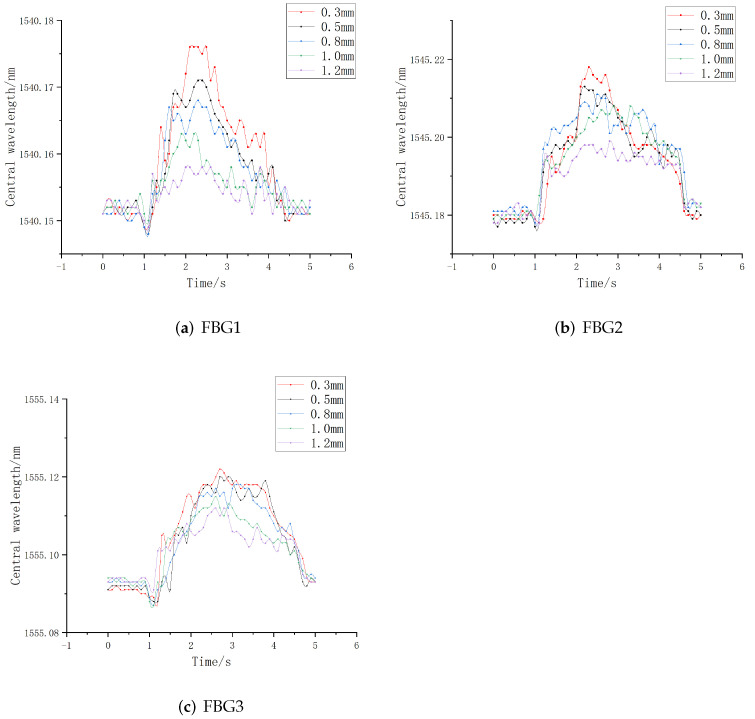
Time-wavelength diagram of FBG.

The sliding perception sensitivity of three FBGs at the embedded depths of 0.3 mm, 0.5 mm, 0.8 mm, 1.0 mm, and 1.2 mm were tested and analyzed. After the sensor was pasted and fixed, the alloy copper block was uniformly sliding on the upper surface of each FBG, and the central wavelength of each FBG was recorded in real time. Thus, the time-wavelength diagram of the FBG under sliding was obtained, as shown in Figure 15.

It can be seen from the above figures that when the embedded depth is 0.3 mm, the overall central wavelength shift of all FBGs is the largest, which is consistent with the simulation results. Then, the FBG sensors with different grating lengths were compared. When the embedded depth is 0.3 mm, the peak values of central wavelength shift of FBG1, FBG2, and FBG3 are 0.027 nm, 0.040 nm, and 0.034 nm, respectively.

The comparison shows that when the embedding depth is 0.3 mm, the overall variation value of FBG2 is the largest, FBG3 is the second largest and FBG1 is the smallest. However, considering the overall structural size of the elastomer, the grating length of 5 mm is too long, and some grating lengths are still exposed to the outside during the encapsulation process, which will affect the detection accuracy in the process of contacting with external objects. Therefore, FBG3 is not suitable for finger sliding experiment. In summary, in the detection of sliding signals, FBG1 and FBG2 have better sensing performance when the embedded depth is about 0.3 mm.

#### 3.2.2. Experimental Analysis of Angle Comparison of Cross-Sensitive Units of Sensing Array Structure

For the problem of sliding detection in different directions, it is necessary to design the FBG sliding tactile sensor as an array structure composed of sensitive units in order to measure physical information, such as the sliding velocity and direction of the object more accurately. In order to obtain the optimal angle of the sensor array unit to detect the sliding direction, this section selects different azimuth angles for sliding sensing experiments.

**Figure 16:**
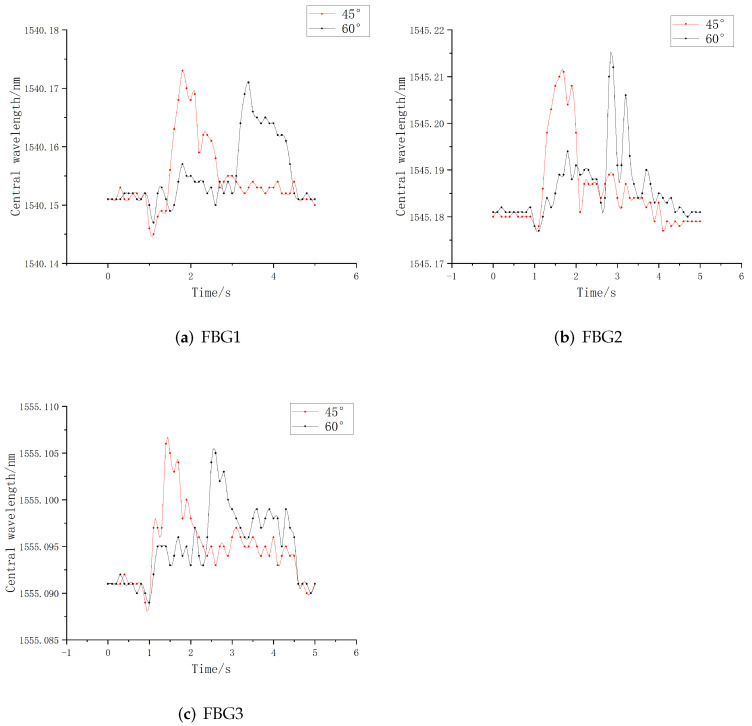
Wavelength relationship diagram for different angles.

Refer to the measurement results of tactile shear force of Qian M. et al. [24], for the shear force in the X direction, the embedding angle of the grating (angle with the Y axis) should be as small as possible. Considering the structural dimensions of the encapsulated elastomer and the convenience of actual calculation, the angles were selected as 40° and 60°, respectively, with the bottom edge as the reference line. The Origin software is used for experimental data analysis to obtain the relationship between central wavelength and time of FBG with different sliding angles, as shown in Figure 16.

It can be seen from the above table that due to the long grating length of FBG3, some gratings are exposed outside the packaging elastomer when tilted, which makes the overall range value accuracy low. When the tilt angle of FBG2 is 60°, its peak value and central wavelength shift are the largest among similar sensors, which shows that FBG2 has good signal sensing ability for sliding tangential strain. When the tilt angle is 45°, the grating section is still partially exposed on the outside, which cannot be completely encapsulated in the elastomer structure. Therefore, for the cross-sensitive unit of the sensing array, we choose the FBG2 with 3 mm grating length as the angle sensor, and determine that the final tilt angle is 60°.

### 3.3. Structural Design and Experimental Analysis of Double-Layer Sliding Tactile Sensor Array

This section will continue to discuss and analyze the distance between the upper sensing unit and the lower cross-sensitive unit to determine the structural size of the final sliding tactile sensing array.

**Figure 17:**
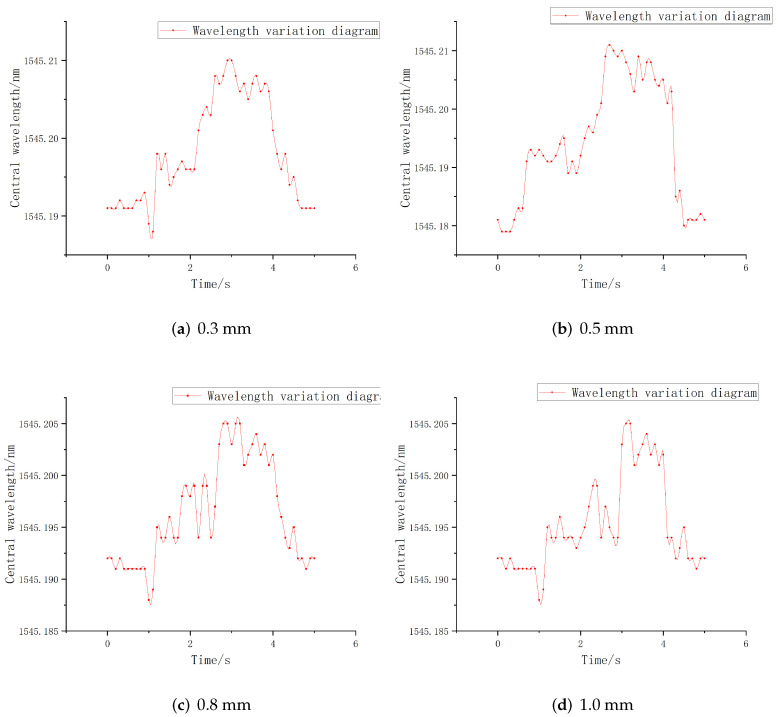
Wavelength-time diagram at different intervals.

In the experiment of upper and lower interval distance, the positions of FBG1 and FBG2 fixed at 0.3 mm from the upper surface are taken as the upper structure, while the FBG with 3 mm grating length is placed at 60° tilt in the lower structure, and the distances between the two structures are selected as 0.3 mm, 0.5 mm, 0.8 mm, and 1.0 mm. The wavelength changes of the lower structure sensor in different interval sliding processes are recorded in real time and imported into the Origin software to draw the central wavelength–time relationship diagram, as shown in Figure 17.

It can be seen from the above figures that when the upper and lower interval is 0.3 mm, the central wavelength drift range of the lower FBG is 0.189–0.210 nm, and the overall change value is 0.22 nm. It reaches the peak at 2.75 s, which is basically consistent with the peak time of the above 60° angle tilt sensor. As shown in Figure 17b, the central wavelength shift range of the lower FBG is 0.189–0.211 nm, and the overall change value is 0.016 nm, reaching the peak at about 3 s. It can be seen from Figure 17c,d that the central wavelength drift ranges of the sensors in the lower sensing unit are 0.190–0.205 nm and 0.190–0.200 nm, respectively, and the change values are 0.016 nm and 0.010 nm. At this time, the overall change value of the central wavelength of the FBG begins to decrease, and the peak arrival time is basically consistent.

In summary, when the interval distance of the lower layer sensor unit is controlled within 0.5 mm, the overall change value is basically controlled at about 0.22 nm, but when the interval value exceeds 0.5 mm, the central wavelength shift range of the FBG sensor begins to decrease. Therefore, the interval distance between the two-layer sensing units shall be 0.5 mm based on comprehensive consideration.

## 4. Experiment and Analysis of Robot Finger Sliding Tactile Sensing Based on FBG

**Figure 18:**
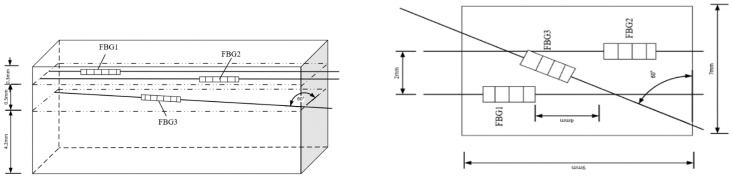
Sensor structure diagram.

As shown in Figure 18, FBGs with grating length of 2 mm and 3 mm were determined as the sensing unit in the upper structure, and their embedded depth is 0.3 mm. In the lower structure, the FBG with a grating length of 3 mm was selected as the sensing unit, and the tilt angle is 60°. The distance between the two layers of sensing units was determined as 0.5 mm. At the same time, in order to better detect the sliding in the central axis direction during the sliding process, a 4 mm interval was set between the upper two FBG segments, so that the slip direction can be judged by the time difference of wave crest during the sliding process. The overall packaged FBG sliding tactile sensing array structure is shown in Figure 18.

### 4.1. Fabrication of FBG Sliding Tactile Sensor

**Figure 19:**
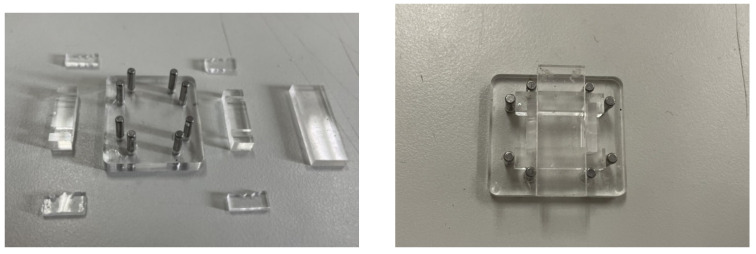
FBG sliding tactile sensor mold.

The sliding tactile sensor designed in this paper is double layer distributed, so PMMA micro-needle die is selected as the manufacturing material, as shown in Figure 19. The whole structure is divided into base, removable three-hole structure, and fixed part. The detachable three-hole structure is divided into the upper part and the lower part. The connection part has three holes to facilitate the placement and packaging of the sensor. At the same time, the left hole is designed as a tapered hole, so that the internal optical fiber will not break when the lower unit is placed. It can be disassembled from top to bottom, effectively ensuring the integrity of the internal elastomer in the demolding process. Cylindrical copper tools are used to fasten around the structure, so that PDMS can be effectively sealed during solidification. After the fiber grating is placed in the hole, it is fixed by the cylindrical part, and then the PDMS mixture is poured from above. After pouring, the excess PDMS mixture is discharged. The sensing mold designed on this basis can not only reduce the pouring process and improve the convenience of the structural demolding, but also has the advantages of repeated use and easy disassembly and cleaning.

### 4.2. Sensing Array Calibration Experiment

(1) Linearity experiment and analysis

**Figure 20:**
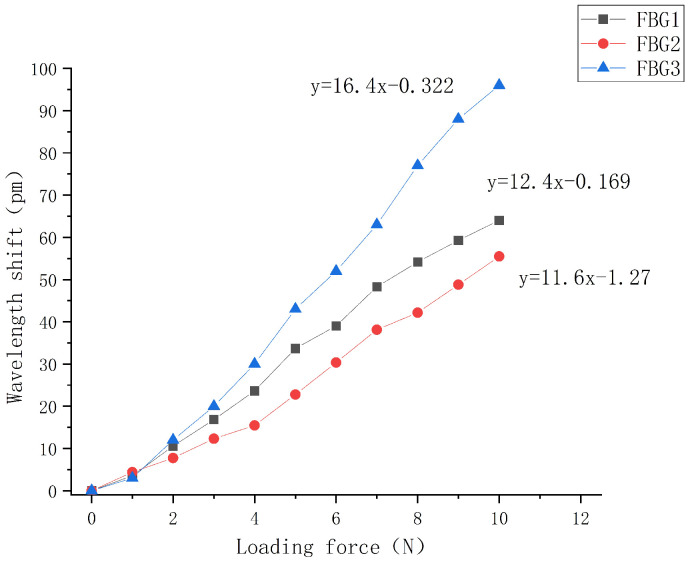
Relationship between central wavelength variation of three sensors and the loading force.

The external uniform load of 0–10 N is gradually applied to the sliding tactile sensor array. The central wavelength shift of each FBG in the sensor array is recorded in real time by the data acquisition software of the computer. The step length is 1 N. The linear fitting diagram is shown in Figure 20.

It can be seen from the figure that the central wavelength of the three sensors in the array increases with the increase in external load, and the overall change is relatively uniform and the central wavelength tends to linear distribution. In the upper structure, the sensitivity of FBG1 (with a grating length of 2 mm) and the FBG2 (with a grating length of 3 mm) can be obtained as 12.4 pm/N and 11.6 pm/N, respectively, according to the fitting curve of wavelength and pressure. The overall linearity of the FBG1 and FBG2 packaged is close as they have the same embedded depth. However, there is a slight deviation from the above mentioned sensitivity of 14.5 pm/N of the two sensors when the buried depth is 0.3 mm. This is because in order to better identify the direction of the sliding sensor during the packaging process, an interval of about 4 mm is set between FBG1 and FBG2, so that the grating segment is not in the center of the elastomer. Under the action of external load, the strain uniformity of the grating section is reduced, resulting in a reduced sensitivity to the change of external load. The central wavelength sensing sensitivity of FBG 3 with the lower grating length of 3 mm is 16.4 pm/N, which has the best linearity in comparison. This is because it is placed at an angle of 60°. When the fiber grating is subject to greater deformation under the load, the two ends of the grating are at the uniform axial strain.

The coefficient of determination $R^{2}$ of the fit goodness of the three sensors in the array are 0.993, 0.995, and 0.997, respectively, indicating that the sensing array structure is relatively stable and can better detect the magnitude of the pressure applied to the upper surface above the sensor, indicating that the sensing array has good linearity and sensitivity.

(2) Repeatability experiment and analysis

**Figure 21:**
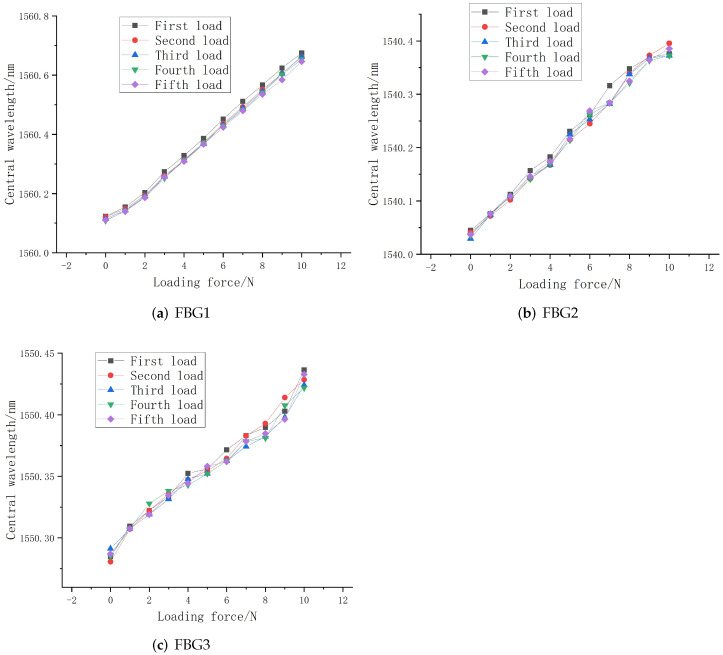
Repeatability fitting curve.

The fabricated sliding tactile sensing array was loaded five times with a distribution of 0–10 N, and the step size was set to 1 N. The characteristic curves of the central wavelength variation of each sensor in the sensing array were plotted, as shown in Figure 21.

It can be seen from the figure that the loading characteristic curves of each sensor in the sensing array have a high degree of overlap, and the overall deviation is controlled within 5 pm without any large deviation, which indicates that the designed sliding tactile sensor array has good repeatability and good fit between the optical fiber and the elastomer. From the analysis of the experimental results, it can be concluded that the repeatability of sensor 1 is significantly higher than that of sensor 2 and sensor 3, which is due to the fact that the sensor with 2 mm grating length can recover from deformation in a short time after the effect of load. It is also evident from the comparison that the different repeatability exhibited by FBG1 and FBG2 at the same embedded depth is also closely related to their respective sensitivities, just like the FBG1 with high sensitivity has better repeatability.

(3) Creep experiment and analysis

**Figure 22:**
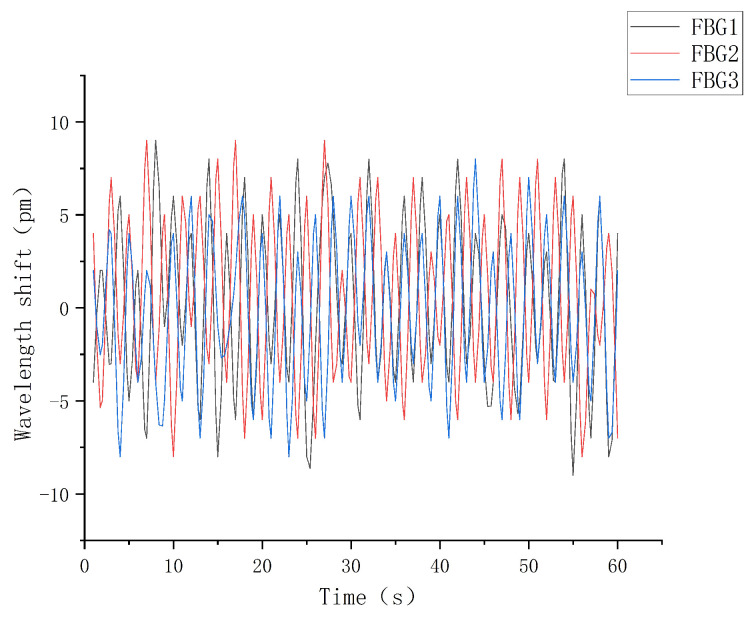
Sensor creep characteristic curve.

The fiber grating sliding tactile sensor array is fixed on the horizontal desktop, the external temperature was controlled to be constant, a constant pressure of 2 N was applied to the upper surface of the elastomer and maintain it for one minute. The central wavelength value of the fiber grating was recorded during this period, and the sampling frequency was set to 1 Hz to collect the shift and fluctuation of the grating central wavelength, and the creep characteristic curve is shown in Figure 22.

As can be seen from the experimental results above, the wavelength variation of each sensor in the sensing array floats steadily around zero scale under a constant external load, and the overall deviation is controlled within 20 pm, which shows that the sensing array has good creep resistance and meets the accuracy requirements. At the same time, as shown in the above figure, the central wavelength shift range of FBG1 is greater than that of FBG2 and FBG3. This is due to the fact that the grating length of FBG1 is 2 mm, while the grating length of FBG2 and FBG3 is 3 mm, which shows that the increasing grating length will make the central wavelength of fiber grating change less.

(4) Directional calibration experiment and analysis

**Figure 23:**
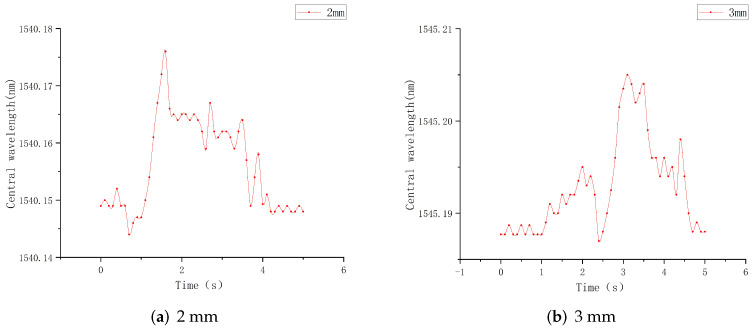
Calibrated wavelength relationship diagram.

The fiber grating sliding tactile sensing array was fixed on a smooth plane, and the intelligent drive trolley was used to pull the alloy copper block sliding on the upper surface of the elastomer at a uniform speed, and the sliding direction was along the x-axis direction. The central wavelength drift values of FBG1 and FBG2 during the sliding process were recorded in real time, and the central wavelength-time relationship of FBG with 4 mm interval was plotted, as shown in Figure 23.

From the experimental results, it can be seen that the two FBG sensors show significantly different moments of rising edge when the object reaches FBG1 and FBG2 at different moments. Taking the forward moving direction as an example, the central wavelength drift range of FBG1 is 0.147–0.170 nm, and the overall change value is 0.023 nm, reaching the peak at 1.5 s, and the peak interval lasts about 1 s. Although the central wavelength drift range of 3 mm FBG2 is 0.187–0.209 nm, and the overall change value is 0.022 nm, reaching the peak at 3 s, and the peak interval lasts about 1 s. During the sliding process, FBG1 first perceived the sliding signal and reached the wave peak when sliding lasted about 1.5 s. The interval between FBG2 and FBG1 is 4 mm, and reached the wave peak at about 3 s. The interval before and after the peak is about 1.5 s. By comparing the different moments when the upper two FBGs in the sensing array reached the rising edge and the wave peak time, the moving direction of the object on the elastic upper surface can be derived. That is, when the copper block slides forward, the wavelength of FBG1 first shifts obviously.

(5) Temperature calibration experiment and compensation analysis

**Figure 24:**
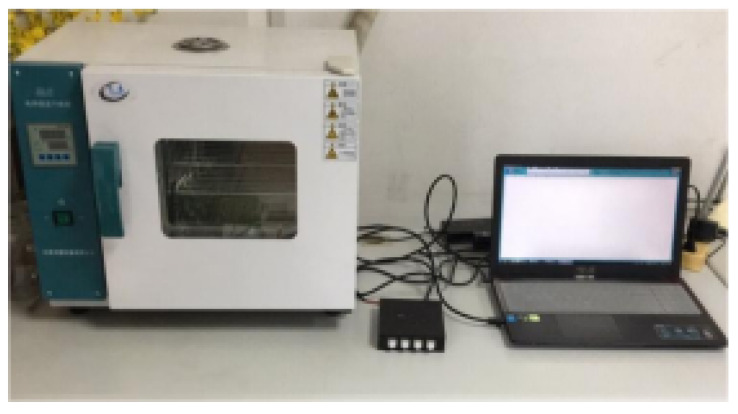
Temperature sensing experimental system.

Because FBG is cross sensitive to strain and temperature, it will be affected by the ambient temperature or the temperature of the object itself when the robot finger grasps the external object. In order to eliminate the influence of temperature on FBG sensor, reduce the error and improve the accuracy of strain measurement, temperature experiment is required. The temperature calibration experimental system consists of fiber grating sensor, incubator, demodulation equipment, and computer, as shown in the Figure 24. FBG sensors with grating lengths of 2 mm and 3 mm were selected for the experiment. After being put into the incubator, the central wavelength shift values at different temperatures were recorded by the demodulator.

**Figure 25:**
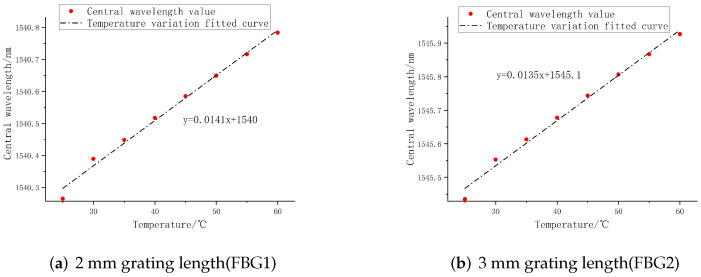
Temperature fitting curve of 25–60 °C.

The temperature range of the incubator was set from 25 °C to 60 °C, and the temperature was measured every 5 °C. The temperature sensing fitting curve of FBG sensor can be obtained as a reference for temperature compensation, and the deviation of temperature fitting linearity of FBG sensors with different grating lengths can be compared. As shown in the Figure 25, FBG1 and FBG2 have high temperature sensitivity coefficients, which are 14.1 pm/°C and 13.5 pm/°C, respectively; the deviations of the central wavelength temperature fit values are 2.9% and 7.14%, respectively, which are controlled within 10%. Among them, the overall temperature fitting linearity of FBG1 is higher than FBG2, and the deviation value is lower, indicating that FBG1 has better temperature sensing characteristics. To sum up, the 2 mm grating length FBG sensor is selected as the temperature compensation reference sensor.

### 4.3. Experiment and Analysis of Robot Finger Grasping Perception

**Figure 26:**
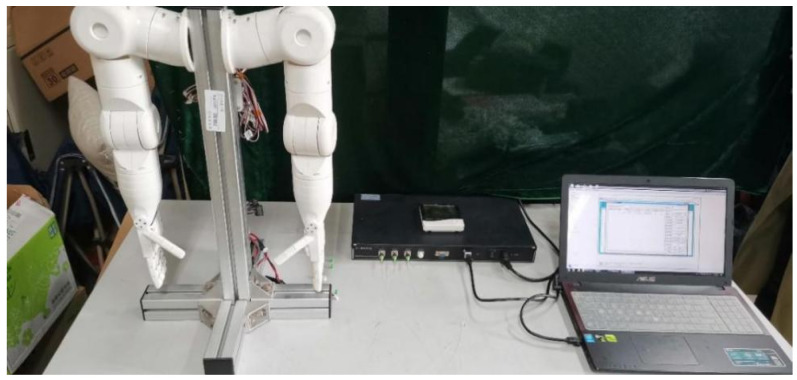
Robot perception test system diagram.

The robot finger grasp perception test experimental system mainly includes demodulation equipment, FBG sliding tactile sensing array, computer, and the robot hand. The perception test system is shown in Figure 26. By controlling the constant external temperature, the experiment has tested two processes of the robot finger contacting and grasping the cylindrical object, and the robot finger shedding and sliding when grasping the cylindrical object, corresponding to the two situations of the robot finger static grasping and object sliding recognition.

**Figure 27:**
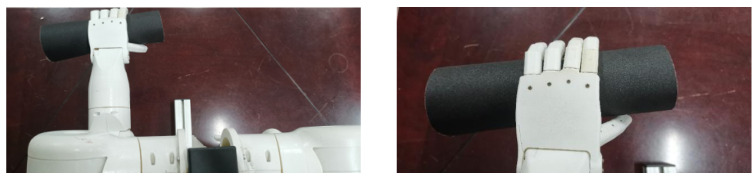
Robot finger grasping diagram.

First, the central wavelength shifts of each sensor in the FBG sliding tactile sensor array was measured in real time by the demodulation equipment; then, the change of the central wavelength of the FBG sensing array was converted into external force to derive the change of its pressure value during the gripping of the robot hand. The physical diagram of the actual gripping experiment of the cylindrical object is shown in Figure 27. In this experiment, the control of robot finger grasping attitude is realized by adjusting the servo control parameters, and the grasping force of robot fingers on cylindrical objects is adjusted.

(1) Experiment and analysis of static grasp perception of robot fingers

**Figure 28:**
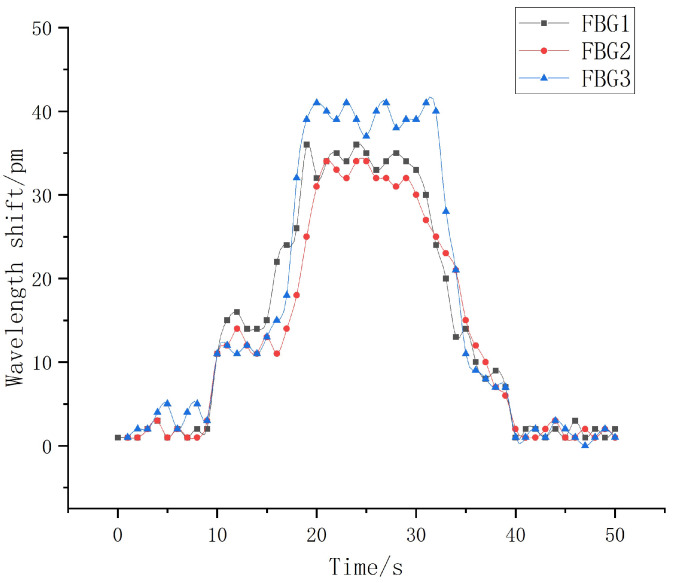
Central wavelength shift diagram of three sensors.

**Figure 29:**
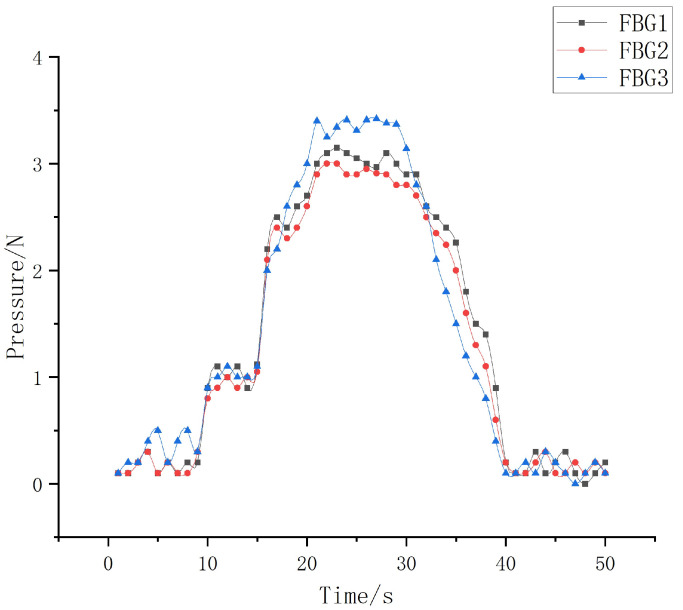
Pressure change diagram of three sensors.

The process of grasping and putting down objects by robot fingers is controlled through the servo control software of the upper computer. At the same time, the sampling frequency of the demodulation device is set to 1 kHz, and the central wavelength drift value when the robot fingers grasp the cylindrical object is collected in real time. By using the Origin drawing software to process and analyze the data, we can obtain the wavelength shift of the sliding tactile sensor array when the robot finger grasps the object, as shown in Figure 28. In order to maintain the stability of the object when the robot finger grasps it, the pressure value applied by the finger to the object at its contact is set to about 3 N. The pressure variation measured by each sensor during the grasping process is shown in Figure 29.

It can be seen from Figure 28 and Figure 29 that the wavelength shift of the robot finger and the pressure change with time in the actual grasping process are basically consistent in the overall trend. At the same time, when the robot fingers grasp the object, the pressure applied on the middle finger can be measured to be about 3.25 N, 3.04 N, and 2.97 N through FBG1, FBG2, and FBG3 in the fiber grating sliding tactile sensor array, which is basically close to the external load of 3 N applied when the robot parameters given. At the same time, FBG3 is subject to greater deformation under load, and its overall induced pressure value has a large deviation, while the pressure induced values of FBG1 and FBG2 are relatively close, which is consistent with the linearity calibration experiment results above. On the whole, the FBG sliding tactile sensor array designed can accurately perceive the whole process of the robot finger grasping the object, which is basically consistent with the simulation results and meets the standard requirements of the experiment.

(2) Experiment and analysis of robot finger dynamic grasping and sliding.

**Figure 30:**
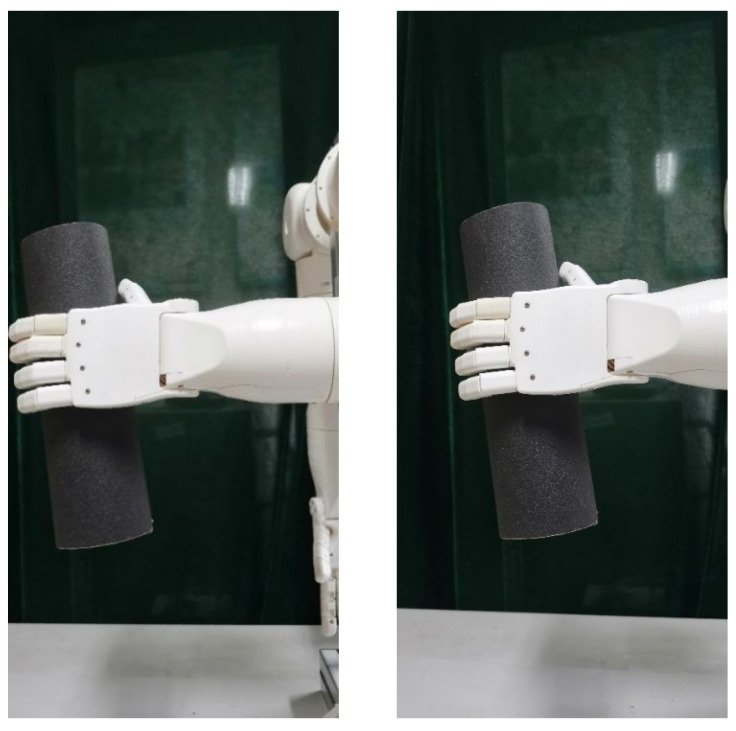
Vertical grasp diagram of robot hand.

**Figure 31:**
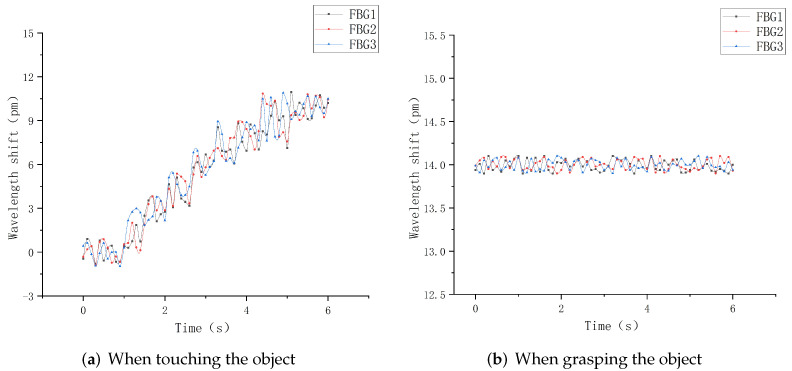
Slip response curve.

**Figure 32:**
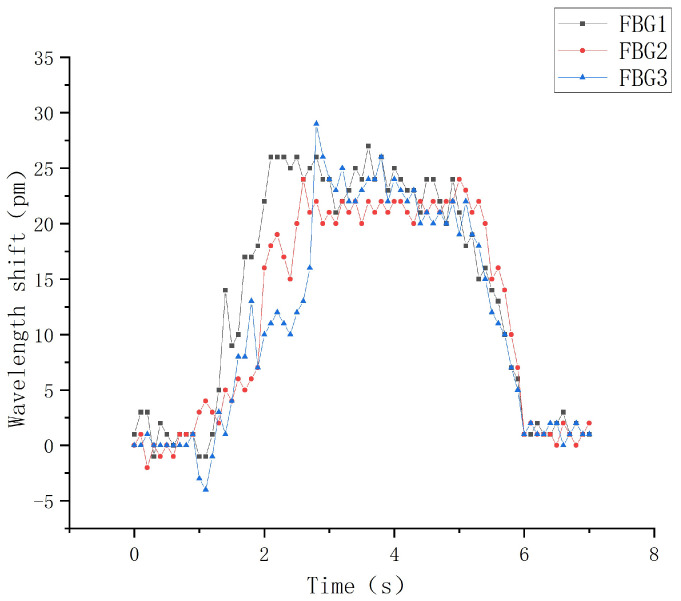
Slip response curve.

This section will carry out experimental analysis on the whole process of the robot finger sliding when it actually grasps an object. Firstly, the robot finger gripping posture was adjusted to vertically grasp a cylindrical object using the servo control interface in the upper computer, as shown in Figure 30. The heavy objects were continuously applied to the cylindrical object to make it start to slip. The sampling frequency of the demodulation device was set to 1 kHz to record the wavelength change of each sensor during the whole process from the beginning of grasping to the end of sliding, and the data were imported into Origin software for processing and analysis to obtain the relationship diagram between the wavelength shift and time, as shown in Figure 31 and Figure 32.

It can be seen from Figure 31a that when the robot finger just touches an object, because its forward pressure and friction force are small, the wavelength shift generated by each sensor in the sensor array is correspondingly small. With the increasing grasp force, the forward pressure and friction force on the sensor array surface are gradually increased, and the central wavelength shift of each FBG is significantly increased. However, Figure 31b shows that the object is in a static state under the grasping force of the robot hand, and there is no relative sliding between the object and fingers, indicating that the object is basically in a stable state, and the central wavelength shift of each sensor at this time is controlled within 3 pm.

Under the gripping state, the cylindrical object starts to slide relatively by applying a certain weight, and the wavelength change value during the sliding process is recorded in time, as shown in Figure 31. It is obvious from the figure that in the actual gripping process when the object starts to slide, all three sensors in the sensing array can better distinguish the three stages of the beginning of sliding, the process of sliding and the end of the sliding. In the process of sliding along the vertical direction, the overall wavelength drift trend of the upper sensors FBG1 and FBG2 is basically the same, and the overall sliding time is controlled at about 5 s. When the sliding starts, the central wavelength of FBG1 starts to increase in a positive direction at the 1 s, reaches the peak range at about 2 s, and ends at about 6 s. The overall sliding range lasts for nearly 4 s, and the change value of the central wavelength shift is 27 pm. Although the central wavelength of FBG2 starts to increase in a positive direction at about 1.9 s, and reaches the peak range at about 2.4 s. The overall sliding process lasts for nearly 3.6 s, and the change value of the central wavelength shift is 25 pm.

In the sliding process, FBG3 is placed at an angle of 60°, so the overall change value is controlled at 18 pm during the vertical sliding sensing process. The central wavelength tends to increase positively at the 2 s, and reaches the peak range at the 2.5 s. The sliding time is ended at about 5.8 s, and the overall sliding signal sensing range is 3.6 s. Meanwhile, according to the comparison with the sliding experimental diagram in the structural design above, the wavelength shift values of each corresponding FBG sensor have basically the same change trend and repeatability, which shows that the FBG sliding tactile sensor array designed in this paper can sensitively perceive the sliding signal of the robot finger, and meet the requirements of dynamic detection. In practical applications, when the target object and the robot finger slide relative to each other, the FBG sliding tactile sensor array can effectively sense the sliding signal, so a threshold value can be set for each sensor in the sensing array. When the wavelength drift generated by the sliding feature value is greater than the threshold value, it can be assumed that the sliding starts to occur. At this time, the signal is timely fed back to the control motor of the robot finger. Thus, the grasping force of robot fingers can be adjusted to further optimize the intelligent grasping ability of robot fingers.

## 5. Conclusions

Based on the fiber grating sensing technology, a double-layer fiber grating sliding tactile sensor is designed for the flexible sliding tactile composite sensing of robot fingers. Firstly, the best embedding depth of FBG in the elastic interior was determined by finite element analysis and pressure experiments. Secondly, the structure of the sensor was optimized by sliding simulation and dynamic experiments. Then, the sensor detection system was built to complete the linearity, repeatability and creep resistance experiments of FBG sliding tactile sensor. Finally, the robot finger actual grasping experiment was carried out to test the sliding tactile perception ability. The results show that:

(1) The designed sensor has good linearity and repeatability, and the experimental results agree with the simulation results. The sensitivity can reach up to 14.5 pm/N, and the response to external load is good; the coincidence degree of loading characteristic curve is high, the deviations are all controlled within 5 pm, and the overall change tends to be linear.
(2) The sensor has good creep resistance characteristic, the wavelength changes stably around the zero scale, and the overall deviation is controlled within 20 pm.
(3) In the robot finger grasping experiment, the sliding tactile sensor has a high fit with the robot finger, and its structure is relatively stable, which shows the feasibility of applying it to the sliding tactile sensor detection of the robot finger surface.
(4) In the robot finger dynamic sliding experiment, the sliding tactile sensing array can effectively sense the sliding signal and meet the needs of dynamic detection.

The experiments in this paper are only for specific robotic fingers and specific grasping scenes. In the future, a large number of grasping test experiments need to be carried out for objects with different friction degrees, shapes and sliding directions. At the same time, we can further explore the impact of different package thicknesses and structures on sensor performance, so as to realize the miniaturization of sensor arrays.

## Figures and Tables

**Table 1:** Relevant parameters of FBG sensor.

Number	Central Wavelength/nm	Grating Length/mm
1	1540.12	2 mm
2	1545.40	3 mm
3	1555.04	5 mm

**Table 2:** Fitting parameters table.

Embedded Depth/mm	Goodness-of-Fit of FBG1	Goodness-of-Fit of FBG2
0.3	0.9915	0.9820
0.5	0.9950	0.9955
0.8	0.9935	0.9925
1.0	0.9935	0.9830
1.2	0.9815	0.9870
2.0	0.9925	0.9905

## Data Availability

The data presented in this study are available on request from the corresponding author.
